# Behavioral Economics, Wearable Devices, and Cooperative Games: Results From a Population-Based Intervention to Increase Physical Activity

**DOI:** 10.2196/games.5358

**Published:** 2016-01-28

**Authors:** Trevor van Mierlo, Douglas Hyatt, Andrew T Ching, Rachel Fournier, Ron S Dembo

**Affiliations:** ^1^ Evolution Health Systems Inc. Toronto, ON Canada; ^2^ Henley Business School University of Reading Greenlands, Henley-on-Thames United Kingdom; ^3^ Rotman School of Managment University of Toronto Toronto, ON Canada; ^4^ Zerofootprint Toronto, ON Canada

**Keywords:** behavioral economics, cooperative games, adherence, compliance, serious games, Superusers, health rewards, internet of things, wearable devices

## Abstract

**Background:**

Health care literature supports the development of accessible interventions that integrate behavioral economics, wearable devices, principles of evidence-based behavior change, and community support. However, there are limited real-world examples of large scale, population-based, member-driven reward platforms. Subsequently, a paucity of outcome data exists and health economic effects remain largely theoretical. To complicate matters, an emerging area of research is defining the role of Superusers, the small percentage of unusually engaged digital health participants who may influence other members.

**Objective:**

The objective of this preliminary study is to analyze descriptive data from GOODcoins, a self-guided, free-to-consumer engagement and rewards platform incentivizing walking, running and cycling. Registered members accessed the GOODcoins platform through PCs, tablets or mobile devices, and had the opportunity to sync wearables to track activity. Following registration, members were encouraged to join gamified group challenges and compare their progress with that of others. As members met challenge targets, they were rewarded with GOODcoins, which could be redeemed for planet- or people-friendly products.

**Methods:**

Outcome data were obtained from the GOODcoins custom SQL database. The reporting period was December 1, 2014 to May 1, 2015. Descriptive self-report data were analyzed using MySQL and MS Excel.

**Results:**

The study period includes data from 1298 users who were connected to an exercise tracking device. Females consisted of 52.6% (n=683) of the study population, 33.7% (n=438) were between the ages of 20-29, and 24.8% (n=322) were between the ages of 30-39. 77.5% (n=1006) of connected and active members met daily-recommended physical activity guidelines of 30 minutes, with a total daily average activity of 107 minutes (95% CI 90, 124). Of all connected and active users, 96.1% (n=1248) listed walking as their primary activity. For members who exchanged GOODcoins, the mean balance was 4,000 (95% CI 3850, 4150) at time of redemption, and 50.4% (n=61) of exchanges were for fitness or outdoor products, while 4.1% (n=5) were for food-related items. Participants were most likely to complete challenges when rewards were between 201-300 GOODcoins.

**Conclusions:**

The purpose of this study is to form a baseline for future research. Overall, results indicate that challenges and incentives may be effective for connected and active members, and may play a role in achieving daily-recommended activity guidelines. Registrants were typically younger, walking was the primary activity, and rewards were mainly exchanged for fitness or outdoor products. Remaining to be determined is whether members were already physically active at time of registration and are representative of healthy adherers, or were previously inactive and were incentivized to change their behavior. As challenges are gamified, there is an opportunity to investigate the role of superusers and healthy adherers, impacts on behavioral norms, and how cooperative games and incentives can be leveraged across stratified populations. Study limitations and future research agendas are discussed.

## Introduction

Opportunities related to behavioral economics [1-2], wearable devices [3-4], tailored evidence-based behavior change tools [5-7], and community support [8-9] are highlighted in health care literature, but their collective integration into real-world interventions are limited in scope. Subsequently, there is a paucity of outcome data, and health economic effects remain largely theoretical.

While the literature does investigate the relationship between exercise and the use of less sophisticated wearable devices, such as pedometers [10-11], there are little, if any, published outcomes on more sophisticated devices (such as Fitbit, Jawbone, or Apple Watch), the use of tracking apps (such as Moves, Runtastic Pedometer, or Pedometer++), or health-tracking platforms (such as Google Fit, MapMyFitness, or GOODcoins).

However, this will soon change. One source identifies over nearly 300 registered clinical trials that are utilizing devices in their protocols [12]. A recent pharma-focused trade publication notes that although in its infancy, wearables are emerging as a multifaceted solution to typical problems in clinical trials [13], and Google is developing a wristband health tracker specifically for the clinical research industry [14]. In relation to physical activity, tailored prescriptions that leverage personalized algorithms and wearables are feasible [15].

These efforts to leverage digital health tools and behavioral incentives [16-20] have escalated in recent years, perhaps due to the growing health care crisis in North America. Costs of medication and treatment non-adherence are estimated to exceed $300 billion each year [21-22], and policy theorists have identified technology’s potential to have an important impact on decreasing costs and increasing intervention efficacy [23-24].

However, researchers are showing concern over high program attrition rates [25], sustainability [26], and the failure of digital health to show impacts at population levels [27-28]. A countermeasure is that increasing amounts of data becoming available, and analysis of specific usage patterns and topologies are becoming more insightful [29-30].

For example, a rule of thumb in digital marketing is the 1% rule, or 90-9-1 principle, which seeks to explain network participatory patterns [31]. The rule states that 90% of network actors observe and do not participate, 9% contribute sparingly, and 1% of actors create the vast majority of new content. This 90%, 9%, and 1% are also known as Lurkers, Contributors, and Superusers, respectively [32].

Since healthy adherers and Superusers tend to be the primary contributors to community-based tools, a concern is that interventions are mainly utilized by healthy adherers, individuals who are already highly engaged in healthy behaviors [33-34]. However, it is also possible that in community-based platforms, Superusers may influence those who are less active.

### Objective

The objective of this preliminary study is to analyze descriptive data from GOODcoins, a self-guided, free-to-consumer engagement and rewards platform incentivizing walking, running and cycling.

### The Intervention

Registered members accessed the GOODcoins platform through PCs, tablets or mobile devices. Following registration, users were encouraged to sync wearable devices such as Jawbone, Fitbit or the Moves App to their profile.

Zerofootprint Software Inc., the Toronto-based Corporation that manages GOODcoins, is a software company that aggregates data from sensors, databases, medical devices, smart electronics and telematics for the purposes of creating evidence and reward-based behavior change. GOODcoins is a social currency that is being awarded to members for achieving activity goals. There is no cost or membership fee, and any individual can join the GOODcoins platform. Cumulative and anonymized data generated from the program is analyzed by Zerofootprint, program sponsors, or academic partners.

### Gamified Group Challenges Measuring Individual Progress

Following registration and the syncing of their wearable device or app, members were encouraged to opt into various gamified group challenges that involved walking, running, or biking.

**Figure 1:**
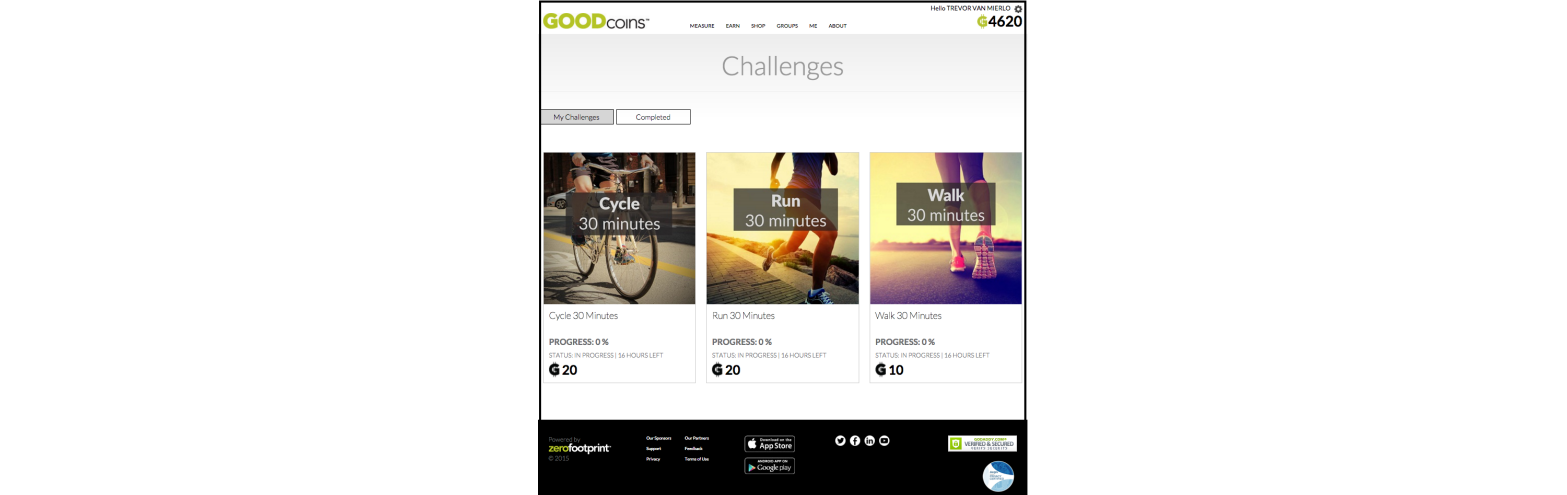
GOODcoins opt-in challenges for November 11, 2015.

Each challenge was unique, and had its own reward structure. GOODcoins (or partner organizations that sponsored a challenge) determined the challenge reward structures. Figure 1 illustrates three specific challenges that members could choose to join on November 11, 2015.

**Figure 2:**
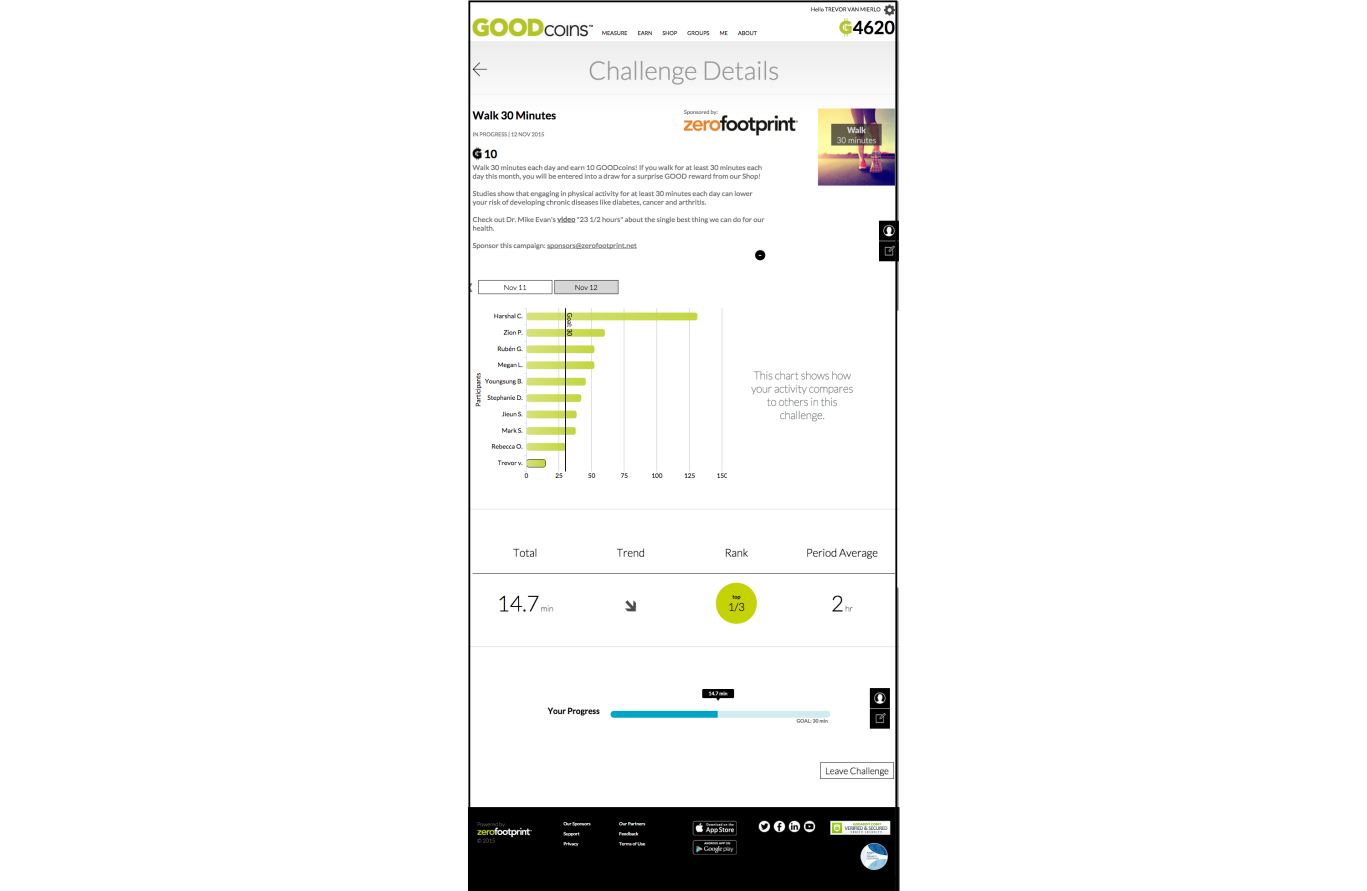
Walk 30 minutes challenge.

Once a member joined a specific challenge, they were able to compare their progress to other GOODcoins members. Figure 2 illustrates the challenge “Walk 30 Minutes”. In this challenge, members were rewarded 10 GOODcoins if they walked 30 minutes each day.

The challenge was gamified as it allowed users to measure their daily progress. Normative feedback allowed members to compare their progress to other members who also opted into the challenge. This is illustrated by the chart to the left of the text “This chart shows how your activity compares to others in this challenge”. The section below the chart outlined the member’s daily progress on an individual level.

**Figure 3:**
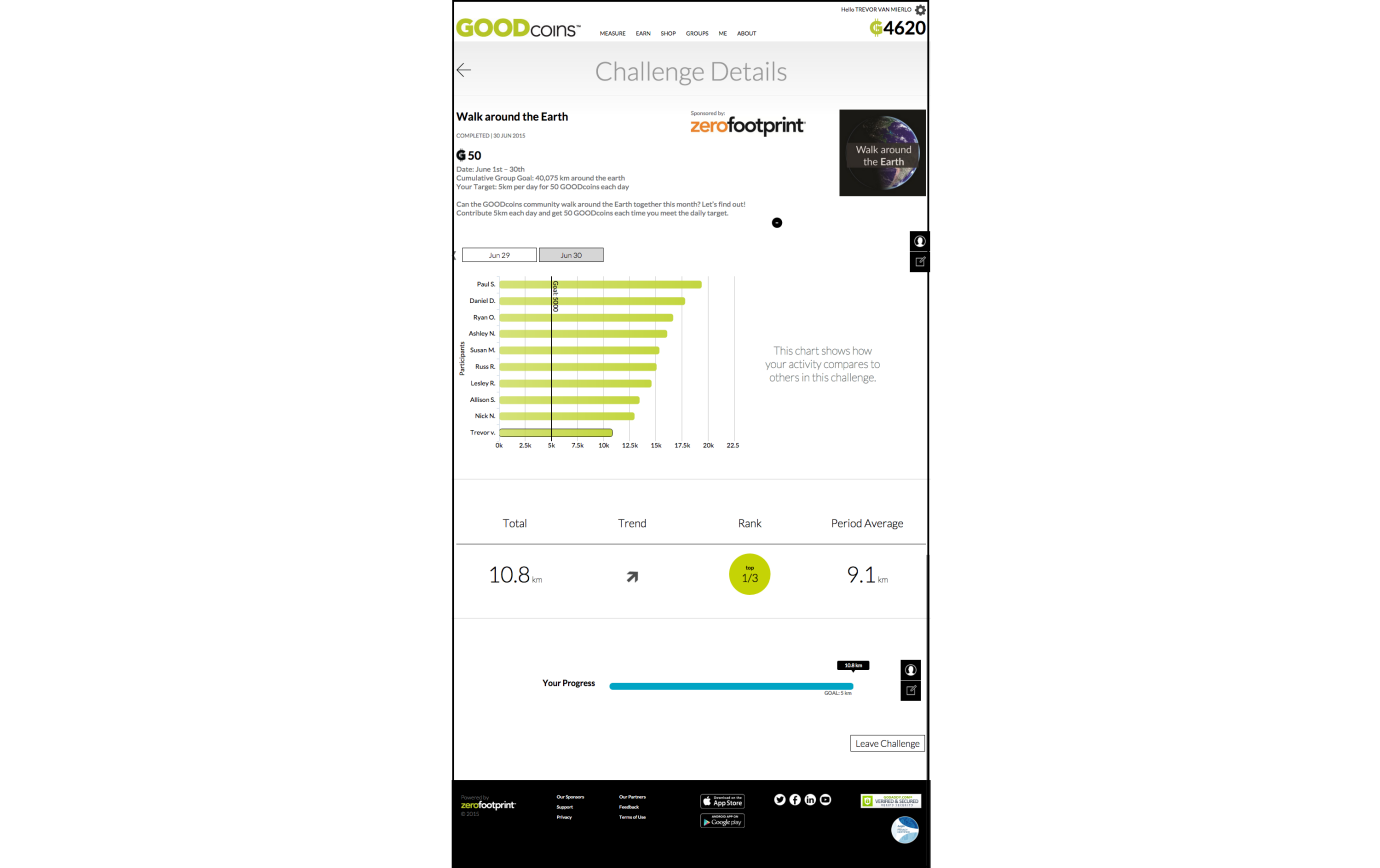
Walk around the earth challenge.

Periodically, new, short-term challenges were offered to the community. Figure 3 illustrates the completed challenge “Walk around the Earth”.

In this challenge, cooperative game theory was utilized to encourage members to individually contribute to a single overall goal. The challenge, offered between June 1, 2015 and June 30, 2015, was initiated by the question: “Can the GOODcoins community walk around the earth together this month? Let’s find out! Contribute each day and get 50 GOODcoins each time you meet the daily targets”.

Each member who accepted the challenge was incentivized by being rewarded 50 daily GOODcoins for walking 5 kilometers per day.

### Sponsored Challenges

**Figure 4:**
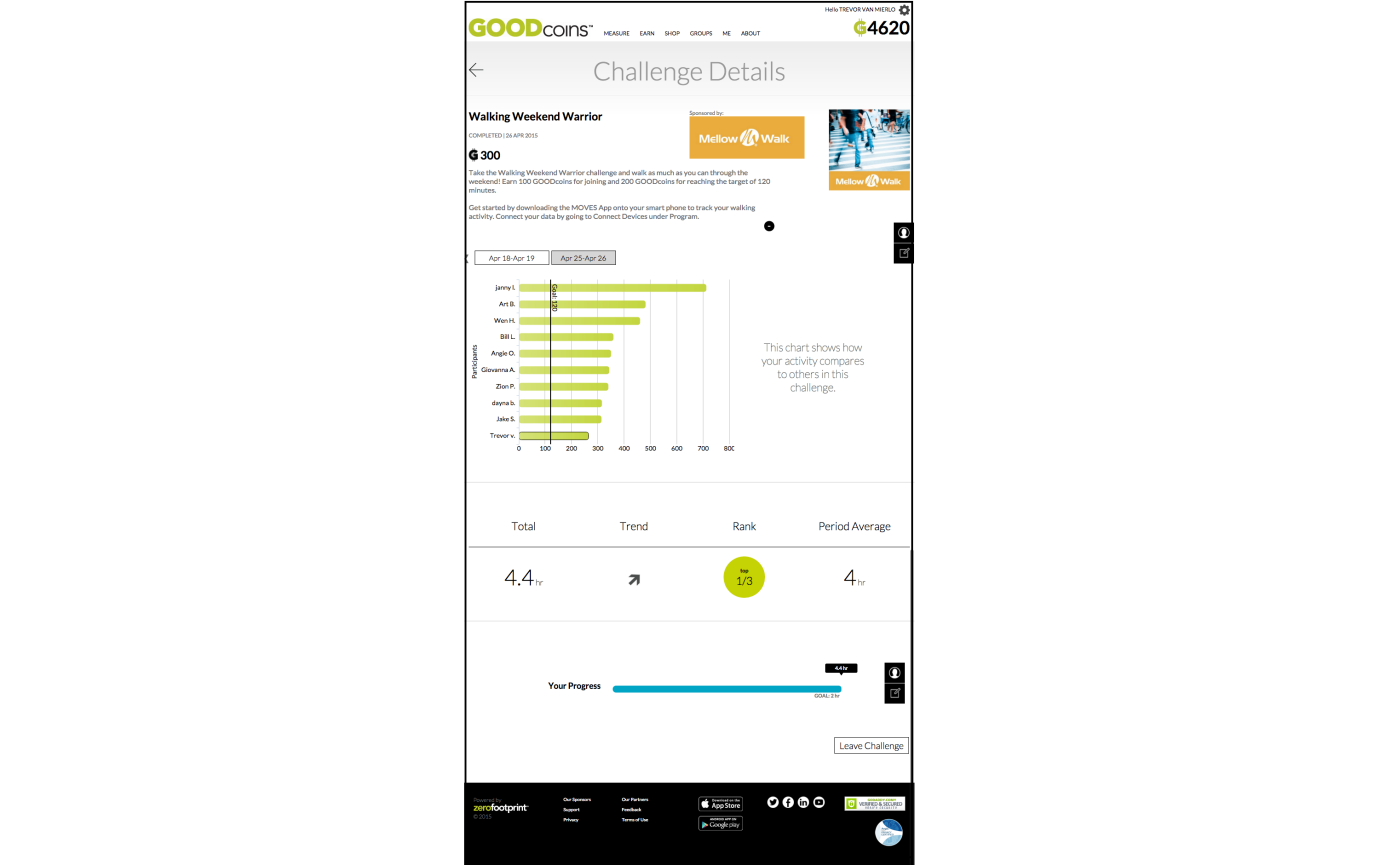
Challenge sponsored by Mellow Walk.

Challenges are sponsored by Zerofootprint, or partner organizations. Figure 4 is an example of a challenge titled “Walking Weekend Warrior”, which was sponsored by Mellow Walk, a Canadian shoe retailer. The challenge was offered over one specific weekend, and members were rewarded with 100 GOODcoins for joining, and 200 GOODcoins for reaching the target of 120 minutes.

### Rewards

**Figure 5:**
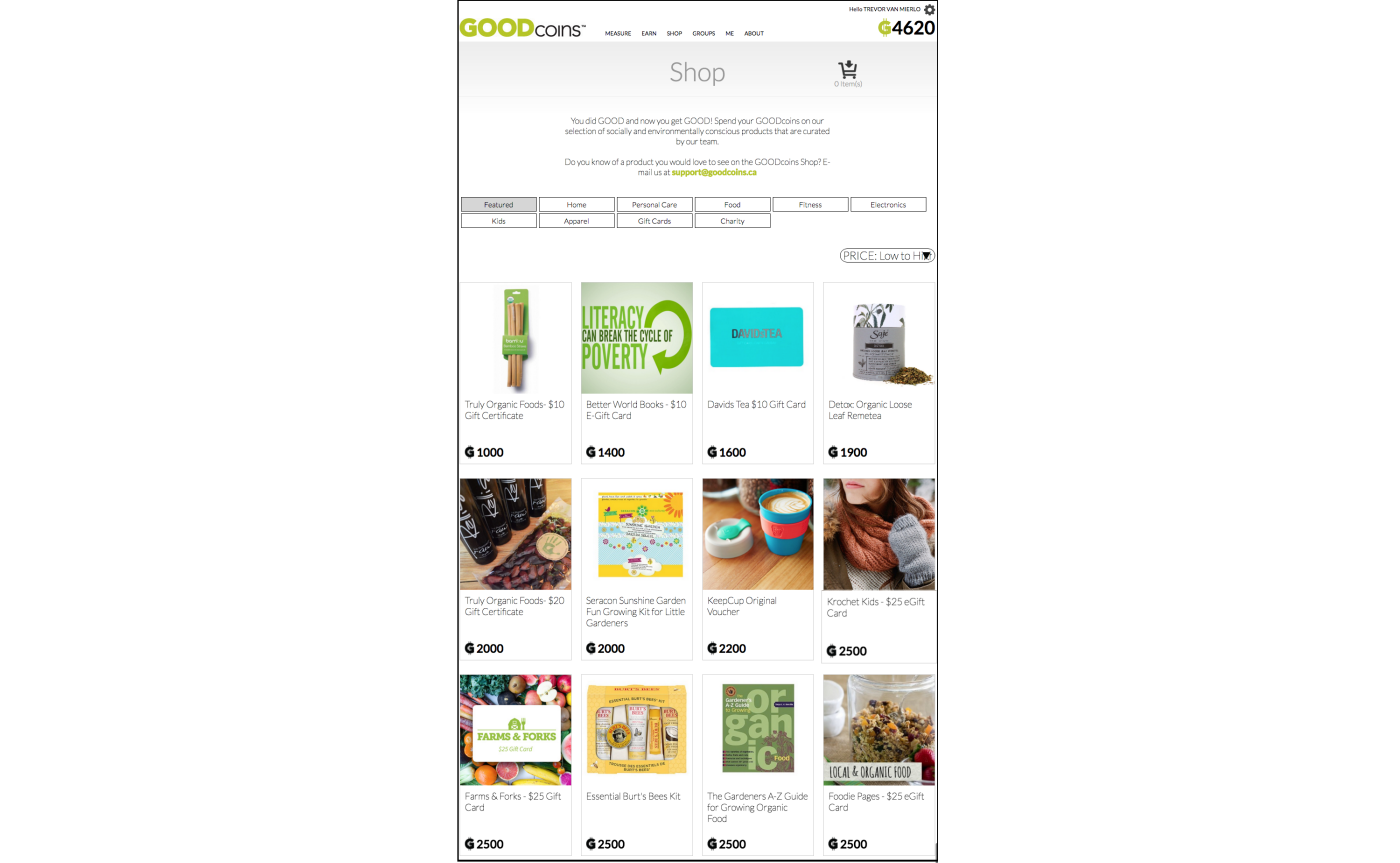
Sample products in the GOODcoins shop.

As members met challenge targets and accumulated GOODcoins, they had the option of redeeming their GOODcoins for planet- or people-friendly products offered in the GOODcoins shop (Figure 5).

All products were curated by the GOODcoins team, and were deemed to be socially and environmentally conscious.

### Anticheat Measures

Although it is not possible to ensure that members, groups of members, or a single member posing as a group of members do not commit fraud, risk is managed in 4 specific ways. First, statistical techniques can be used to detect whether member movements fall within realistic ranges. Movement that is not within a realistic range is flagged. Second, internal staff reviews challenges and challenge completion rates. Third, redemption rates in the GOODcoins shop are monitored. Finally, members of the GOODcoins community have the opportunity to contact GOODcoins directly if they observe atypical behavior in gamified group challenges.

## Methods

The reporting period was December 1, 2014 to May 1, 2015. Descriptive self-report data were analyzed using MySQL and MS Excel. All member data are self-report. Outcome data in this study was obtained from the GOODcoins custom SQL database.

At registration all members consented to the use of their data for research or commercial purposes. Data collection procedures adhered to Canadian privacy guidelines [35].

Prior to analysis, data was scrubbed of test cases, and de-identified. Typical of digital health studies based on retrospective databases which are free of personally identifiable information, the authors deemed the study exempt from formal, ethical review.

## Results

### Overall Findings

The study period includes data from 1298 users who were connected to an exercise-tracking device. Females consisted of 52.62% (683/1298) of the population, 33.74% (438/1298) were between the ages of 20-29, and 24.81% (322/1298) were between the ages of 30-39. Canadians comprised 89.45% (1161/1298) of the sample (Table 1).

**Table 1:** Demographic characteristics of GOODcoins members.

General characteristics		n (%)
Total population		4342 (100.0)
Total population using health care challenges		1298 (29.9)
**Gender**		
	Female	683 (52.6)
	Male	557 (42.9)
	Unknown	58 (4.5)
**Age**		
	19 and under	70 (5.4)
	20-29	438 (33.7)
	30-39	322 (24.8)
	40-49	254 (19.6)
	50-59	106 (8.2)
	60 and above	30 (2.3)
	Unknown	78 (6.0)
**Nationality**		
	Canadian	1161 (89.4)
	American	90 (6.9)
	Other	47 (3.6)

Table 2 outlines activity recorded from members’ wearable devices. Over 77% of connected and active members (1006/1298) met daily recommended physical activity guidelines of 30 minutes, with a total daily average activity of 107 minutes (95% CI 90-124).

Slightly over 96% of connected and active users (1248/1298) engaged in walking as their primary activity, versus 1.54% who preferred running (20/1298), or 2.31% who preferred cycling (30/1298).

**Table 2:** Activity.

Primary activity	Percentage of user engagement, n (%)
Walking	1248 (96.14)
Running	20 (1.54)
Cycling	30 (2.31)

Of members who exchanged GOODcoins, the mean balance at time of redemption was 4,000 (equivalent to approximately US $40) (95% CI 3850-4150). Over 50% (61/122) of redemptions were for fitness or outdoor products, while 4.1% (5/122) were for food-related items (Table 3).

**Table 3:** Redemption categories.

Redemption categories	n (%)
Food	5 (4.1)
Apparel	14 (11.6)
Home and Garden	15 (12.4)
Personal Care	26 (21.5)
Fitness/Outdoor	61 (50.4)

**Figure 6:**
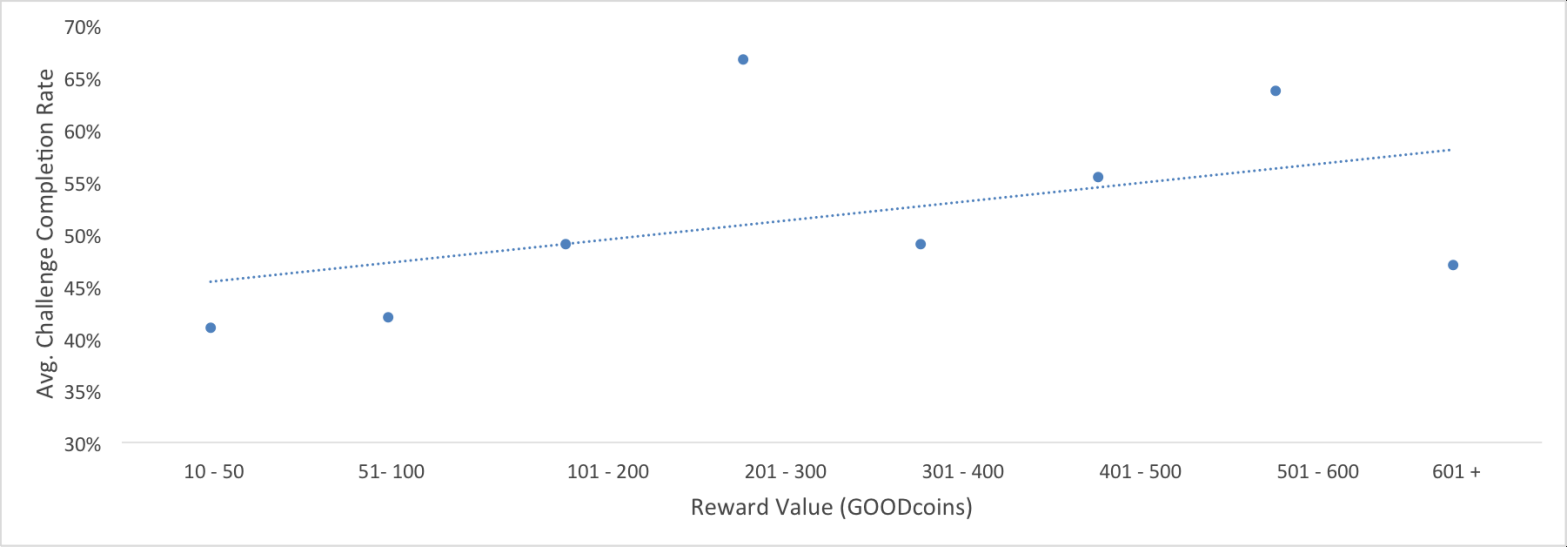
Relationship between reward value & challenge completion rate.

Participants were most likely to complete challenges when rewards were between 201-300 GOODcoins (Table 4 and Figure 6).

**Table 4:** Challenge completion rate.

GOODcoins value	Average challenge completion rate, %
10-50	41
51-100	42
101-200	49
201-300	67
301-400	49
401-500	55
501-600	64
601+	47

## Discussion

### Principal Findings

This analysis is observational, and its purpose is to form a baseline for future research in this rapidly emerging field. Results indicate that challenges and incentives may be effective for connected and active members, and may play a role in achieving daily recommended activity guidelines.

Members were generally female (52.62%, 683/1298) and under the age of 40. Walking was the primary activity (96.14%, 1248/1298), and 50.4% of rewards (61/121) were exchanged for fitness or outdoor products. More detailed demographic and psychographic data could assist in the development of profile utilization patterns, motivations, and impact on overall health behaviors.

To achieve health benefits, Canadian adults aged 18-64 should accumulate at last 150 minutes of moderate- to vigorous-intensity aerobic activity per week in bouts of 10 minutes or more [36]. It is encouraging to note that 77.50% (1006/1298) of connected and active members were physically engaged for at least 30 minutes per day, with a total daily average activity of 107 minutes (95% CI 90-124).

Participants were most likely to complete challenges when rewards were between 201-300 GOODcoins (67% completion rate). However the challenge completion rate was 64% when rewards were between 501 and 600 GOODcoins, 55% between 401 and 500 GOODcoins, and 47% when GOODcoin rewards were over 600. This lack of detectable trends warrants investigation into how rewards and incentives are positioned. Researchers may wish to apply models familiar to economics and finance such as hyperbolic discounting, operant conditioning, or matching law.

Yet to be determined is whether members were already physically active at time of registration and are representative of healthy adherers, or were previously inactive and were incentivized to change their behavior.

As outlined in Figure 1, challenges are gamified, and recent health studies have illustrated positive effects from group-based challenges [17-18]. Future research should also investigate the role of Superusers and healthy adherers, and their impact on behavioral norms.

### Strengths and Limitations

A strength of this study is that participants belong to a naturalistic, self-seeking population that may be representative of digital patients who seek to participate in rewards programs. However, this same strength may also be interpreted as a weakness as the program may primarily attract healthy adherers. It will also be important for future research to analyze 3, 6 and 12-month trends to determine increases or decreases in individual physical activity levels, and assess if subjects are reaching incentives and goals.

An additional strength is the inclusion of real-time data from wearable devices. Data are continually synced from devices to the GOODcoins platform, so it would be difficult for users to manipulate results.

Absent from this analysis are details examining the potential relationships between number of participants in a specific type of challenge, variance of completion rates, and reward value. Future research should consider the optimization of these relationships through the lens of economic models such as hyperbolic discounting or pooling, or behavior change strategies such as normative feedback or motivational interviewing.

An important limitation is that all connected wearables track walking (steps), however only a few had the capability of calculating movement associated with running or cycling. Therefore, differences between walking, running and cycling should be interpreted with caution.

### Conclusions

Challenges and incentives may be effective for connected and active members, and may play a role in achieving daily recommended activity guidelines. Data from rewards-based activity programs can give insights into theoretical constructs related to behavioral incentives, gamification, and strategies associated with cooperative games. Further research examining demographic and psychographic characteristics of rewards-program members, program efficacy rates, and the stratification of member-types is required.
